# Supplementary material for: The Stress-Inducible BCL2A1 Is Required for Ovarian Cancer Metastatic Progression in the Peritoneal Microenvironment
Source: Cancers (Basel). 2021 Sep 12;13(18):4577. doi: 10.3390/cancers13184577 (PMC8469659; doi:10.3390/cancers13184577)
Supplement: Supplementary file 1 [file cancers-13-04577-s001.zip › Supplementary Fig. S1.pdf]

Supplementary Fig. S1

Dominant signaling pathways	Gene hits
Wnt signaling pathway(P00057)	CDH10, CDH13, HDAC3, GNG4
Inflammation mediated by chemokine and cytokine signaling pathway(P00031)	ALOX5AP, CCL5, IL-8, GNG4
Apoptosis signaling pathway(P00006)	BCL2A1, MAP4K1
Heterotrimeric G-protein signaling pathway-Gi alpha and Gs alpha mediated pathway(P00026)	GRM1, GRK1, GNG4

Gene ontology analysis of the whole human genome expression array results using genes with upregulation or downregulation >2 folds from a panel of gynaecological cancer cell lines treated by Hypoxia (0.5% O2, 24 h) and normoxia.
