# Supplementary material for: The Stress-Inducible BCL2A1 Is Required for Ovarian Cancer Metastatic Progression in the Peritoneal Microenvironment
Source: Cancers (Basel). 2021 Sep 12;13(18):4577. doi: 10.3390/cancers13184577 (PMC8469659; doi:10.3390/cancers13184577)
Supplement: Supplementary file 1 [file cancers-13-04577-s001.zip › Supplementary Fig. S2.pdf]

Supplementary Fig. S2

Model ID	Model Name	Score	Relative Score	Start	End	Strand	Predicted Site Sequence
MA0107.1	RELA	7.97	0.808987952	256	265	-1	GTGAATTTCA
MA0107.1	RELA	10.52	0.875171483	542	551	1	GGGGATTTAC
MA0107.1	RELA	8.837	0.831675879	543	552	1	GGGATTTACC
MA0107.1	RELA	9.531	0.849836687	1330	1330	-1	TGGAATTTCT

Analyzed by JASPAR (http://jaspar.genereg.net/)

The binding site sequence of RELA on the promoter sequence of BCL2A1 was analyzed by JASPAR online database (http://jaspar.genereg.net/). Results revealed that 4 putative binding sites of RELA are localized in the promoter region of BCL2A1.
