# Supplementary material for: The Stress-Inducible BCL2A1 Is Required for Ovarian Cancer Metastatic Progression in the Peritoneal Microenvironment
Source: Cancers (Basel). 2021 Sep 12;13(18):4577. doi: 10.3390/cancers13184577 (PMC8469659; doi:10.3390/cancers13184577)
Supplement: Supplementary file 1 [file cancers-13-04577-s001.zip › Supplementary Fig. S3.pdf]

Supplementary Fig. S3

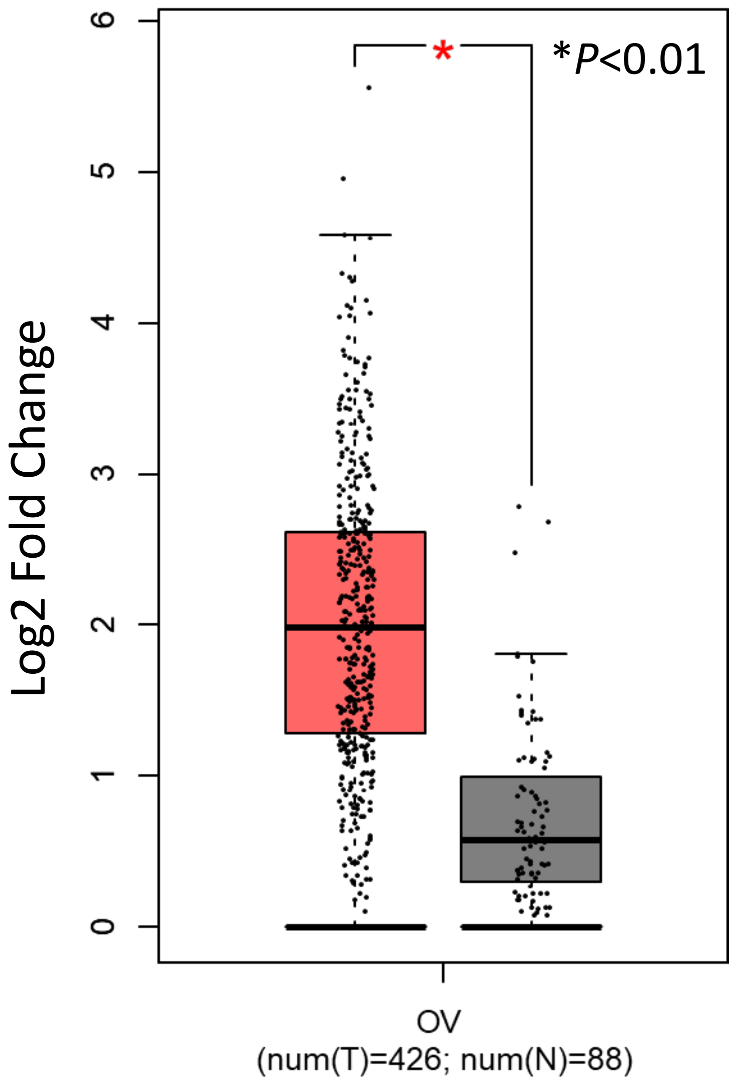

The expression of BCL2A1 in ovarian cancers (N=426) when compared with the normal ovaries (N=88). The data was obtained from TCGA and GTEx, and analyzed by GEPIA (www. http://gepia.cancer-pku.cn). OV=Ovarian cancer. *P<0.01.
