# Supplementary material for: The Stress-Inducible BCL2A1 Is Required for Ovarian Cancer Metastatic Progression in the Peritoneal Microenvironment
Source: Cancers (Basel). 2021 Sep 12;13(18):4577. doi: 10.3390/cancers13184577 (PMC8469659; doi:10.3390/cancers13184577)
Supplement: Supplementary file 1 [file cancers-13-04577-s001.zip › Supplementary Fig. S4.pdf]

Supplementary Fig. S4

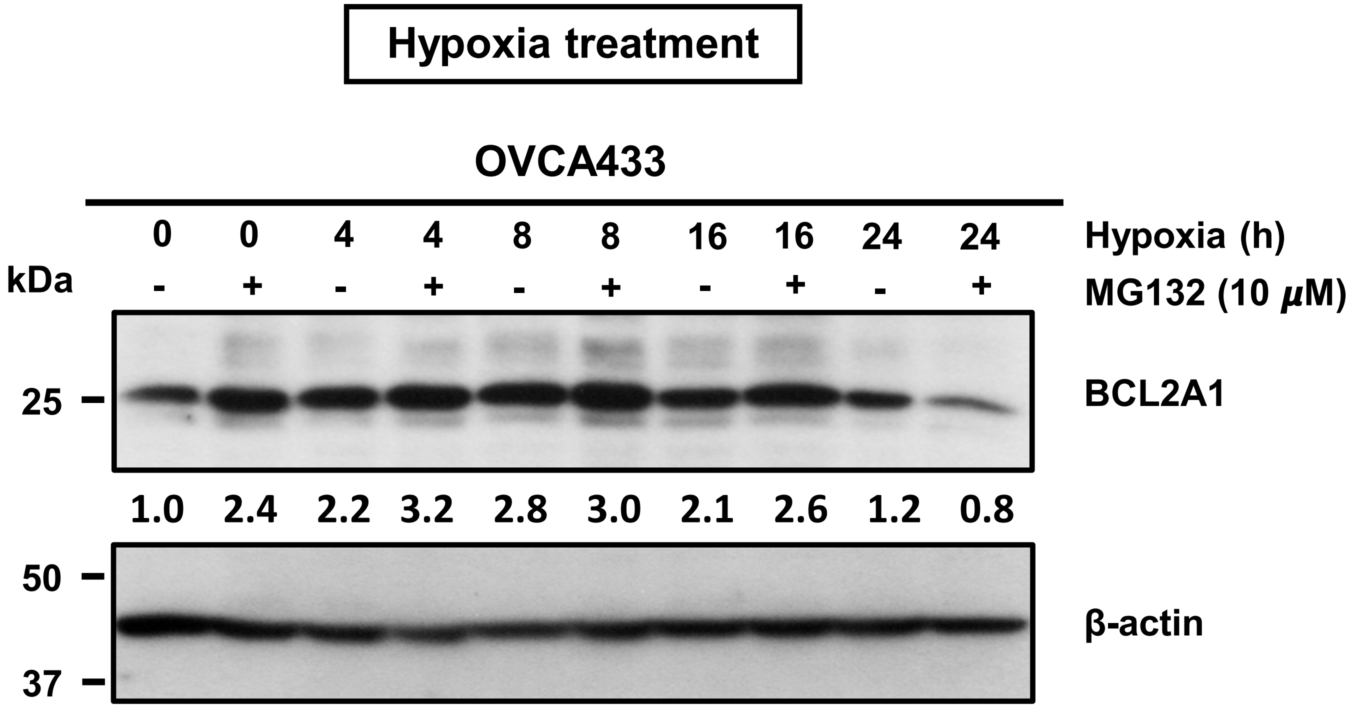

Western blot analysis showed the different protein level of BCL2A1 in OVCA433 treated with or without MG132 (10μg) upon hypoxia (0.5% O2) treatment with various time points .
