# Supplementary material for: The Stress-Inducible BCL2A1 Is Required for Ovarian Cancer Metastatic Progression in the Peritoneal Microenvironment
Source: Cancers (Basel). 2021 Sep 12;13(18):4577. doi: 10.3390/cancers13184577 (PMC8469659; doi:10.3390/cancers13184577)
Supplement: Supplementary file 1 [file cancers-13-04577-s001.zip › Supplementary Fig. S5.pdf]

Supplementary Fig. S5

OVCA433

BCL2A1 overexpression

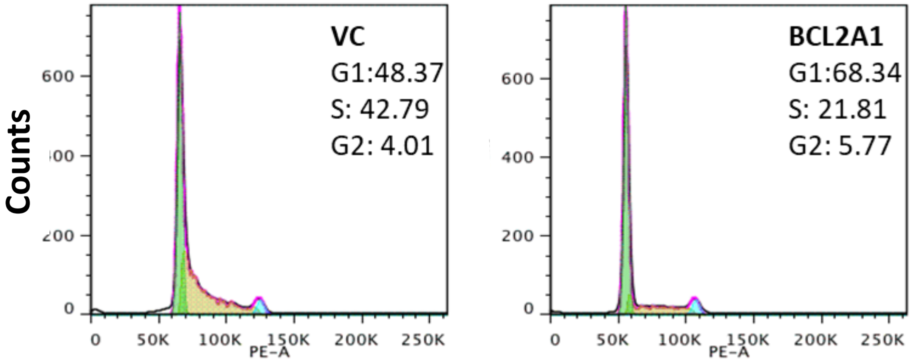

OVCA433

BCL2A1 knockdown

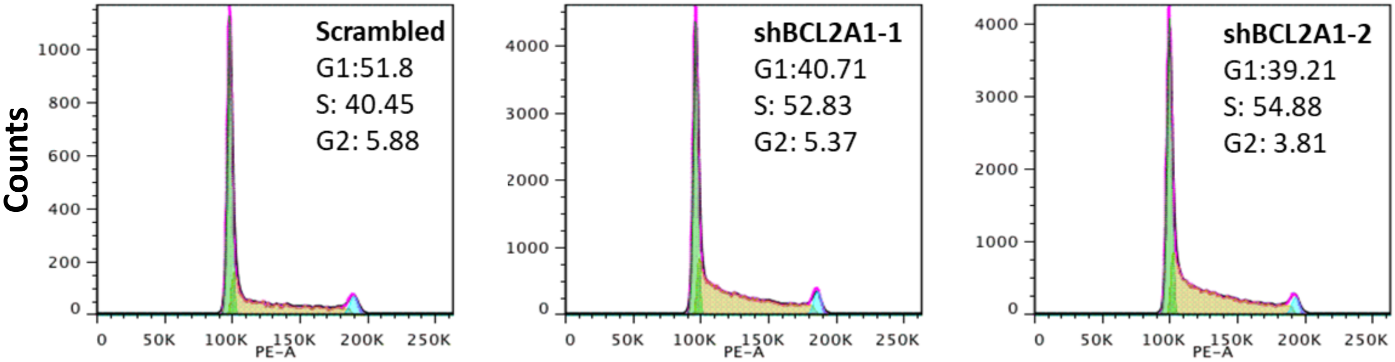

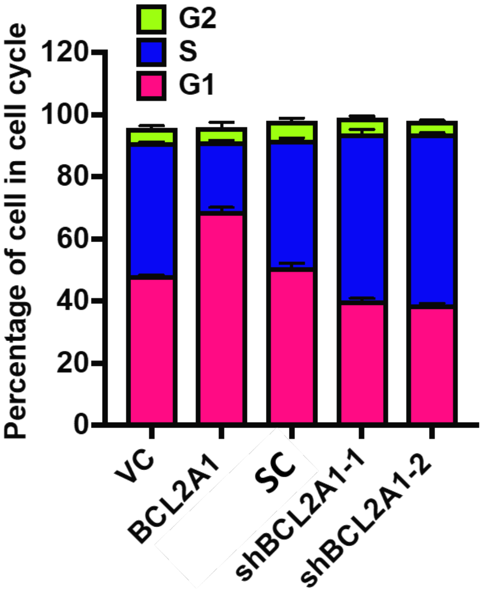

(*Upper*) Flow cytometry cell cycle analysis demonstrated that BCL2A1 overexpressing cells BCL2A1) exhibit higher level of G1-phase arrest compared to vector control cells (VC) in OVCA433. In contrast, knockdown of BCL2A1 (shBCL2A1-1 and shBCL2A1-2) exhibits less G1-phase cell arrest compared to the scrambled Control. (*Lower)* Comparison of cell cycle distribution of cells with BCL2A1 overexpression or knockdown.
