# Supplementary material for: The Stress-Inducible BCL2A1 Is Required for Ovarian Cancer Metastatic Progression in the Peritoneal Microenvironment
Source: Cancers (Basel). 2021 Sep 12;13(18):4577. doi: 10.3390/cancers13184577 (PMC8469659; doi:10.3390/cancers13184577)
Supplement: Supplementary file 1 [file cancers-13-04577-s001.zip › Supplementary Fig. S6.pdf]

Supplementary Fig. S6

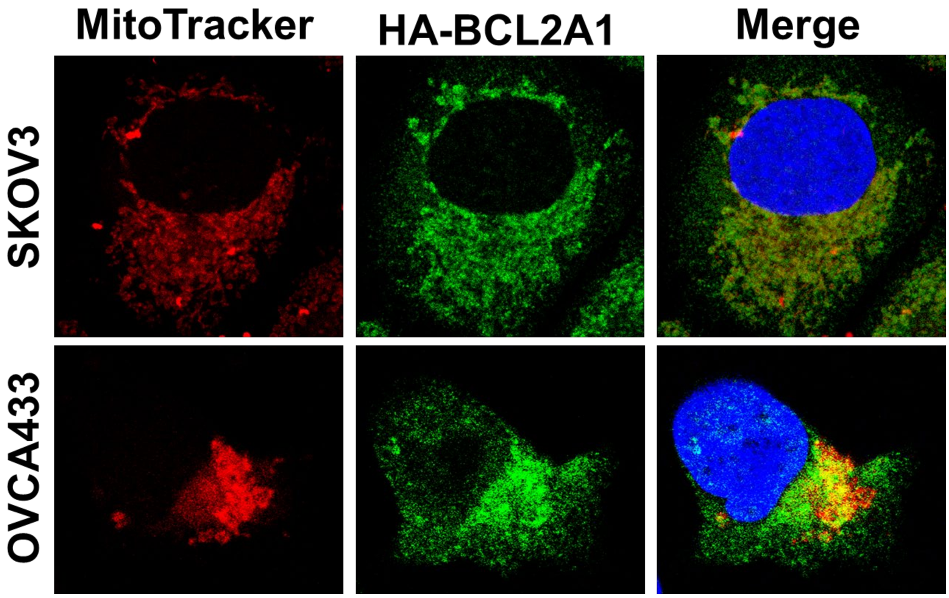

Representative images showing BCL2A1 is localized on mitochondria of ovarian cancer cells. The HA-BCL2A1 plasmid was transient transfection into SKOV3 and OVCA433 cells. After 24 h, ovarian cancer cells were fixed and stained with MitoTracker® (Invitrogen) (Red), anti-HA (Green) and Dapi (Blue). The immunofluorescent images were captured by using confocal microscopy (Nikon).
