# Supplementary material for: The Stress-Inducible BCL2A1 Is Required for Ovarian Cancer Metastatic Progression in the Peritoneal Microenvironment
Source: Cancers (Basel). 2021 Sep 12;13(18):4577. doi: 10.3390/cancers13184577 (PMC8469659; doi:10.3390/cancers13184577)
Supplement: Supplementary file 1 [file cancers-13-04577-s001.zip › Supplementary Fig. S7.pdf]

Supplementary Fig. S7

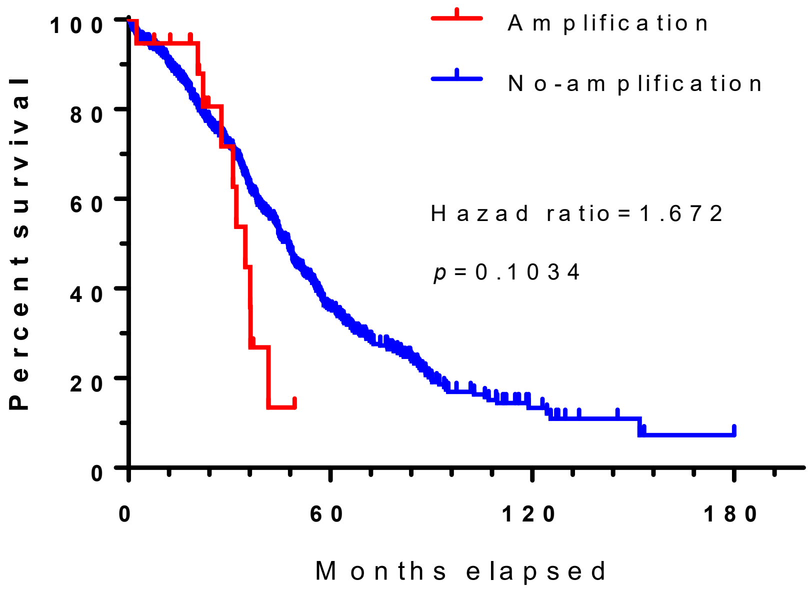

Amplification of BCL2A1 is associated with lower survival ability in ovarian cancer patients. The BCL2A1 gene copy number data together with the patient survival data of ovarian serous cancer (TCGA, 2011) were downloaded from cBioPortal (http://www.cbioportal.org/). Kaplan-Meier survival analysis was performed based on the BCL2A1 amplification showed that the amplification of BCL2A1 was associated with lower survival ability, with a hazard ratio of 1.672, and a log-rank test p-value of 0.1034.
