# Supplementary material for: The Stress-Inducible BCL2A1 Is Required for Ovarian Cancer Metastatic Progression in the Peritoneal Microenvironment
Source: Cancers (Basel). 2021 Sep 12;13(18):4577. doi: 10.3390/cancers13184577 (PMC8469659; doi:10.3390/cancers13184577)
Supplement: Supplementary file 1 [file cancers-13-04577-s001.zip › Supplementary Table S1_CRISPR_sgRNA.pdf]

Supplementary Table S1

In vivo ubiquitination assays

The pcDNA-HA(Ub)8- and BCL2A1-DKK (OriGene)-expressing plasmids were transiently cotransfected into OVCA433 cells. Twenty-four hours after transfection, the cells were treated with MG132 (10 μg) and cultured under hypoxic or normoxic conditions. Anti-DDK (OriGene) was used to pull down ubiquitin-HA from the cell lysates. Magnetic beads conjugated to protein G from the Dynabeads® Protein G kit (Invitrogen) (50 μL) were added to each sample and incubated at 4°C for 2 h. A microcentrifuge tube containing the CoIP protein samples was placed in the DynaMag magnet (Life Technologies) for one minute, and the supernatant was removed. The bead pellets at the bottom were washed with 1 × washing buffer 4 times and 1 × PBS. After removing the washing PBS in the last step, 100 μL of 2 × loading buffer was added to the bead pellets and mixed well by vortexing. The samples were boiled at 95°C for 10 minutes to denature and elute the coimmunoprecipitated proteins from the beads. Twenty microliters of the supernatants of the CoIP products were used for further immunoblotting analysis. Fifteen microliters of cell lysate mixed with an equal volume of 2 × loading buffer was also prepared for immunoblotting.

The sequences of sgRNAs for gene knockout plasmids of HIF-1α and BCL2A1 :

1. **sgRNAs for HIF-1α knockout plasmid:**

sgRNA1 : 5′-GAACTCACATTATGTGGAAG-3′

sgRNA2: 5′- ACAGTAACCAACCTCAGTGT-3′

2. sgRNAs for BCL2A1 knockout plasmid:

sgRNA1 : 5′-GAGTTCATAATGAATAACAC-3′

sgRNA2: 5′- TTGAAGACGGCATCATTAAC-3′
